# Diaphyseal Femur Fractures in Children and Adolescents—Opportunities and Limitations of the ESIN Technique

**DOI:** 10.3390/jcm11247345

**Published:** 2022-12-10

**Authors:** Miriam Kalbitz, Andreas Fischer, Birte Weber, Benjamin Mayer, Ina Lackner, Jochen Pressmar

**Affiliations:** 1 Department of Trauma and Orthopedic Surgery, University Hospital Erlangen, Friedrich-Alexander University Erlangen-Nuremberg, 91054 Erlangen, Germany; 2 Department of Traumatology, Hand-, Plastic- and Reconstructive Surgery, Center of Surgery, University Hospital Ulm, 89081 Ulm, Germany; 3 Department of Trauma, Hand and Reconstructive Surgery, Goethe University of Frankfurt, 60590 Frankfurt am Main, Germany; 4 Institute for Epidemiology and Medical Biometry, Ulm University, 89075 Ulm, Germany

**Keywords:** diaphyseal femur fracture, child, adolescent, ESIN, AO-PCCF

## Abstract

Background: Elastic stable intramedullary nailing (ESIN) is the gold standard for non-overweight children aged 6–12 years. However, the complication rate using elastic stable intramedullary nailing is considerably high. Nevertheless, the question arises of whether the indication for elastic stable intramedullary nailing therapy can be extended and which factors must be taken into account when determining the indication. Methods: A retrospective chart review of patients <18 years admitted with diaphyseal femur fracture at a Level I Trauma Center in Germany between 2005 and 2017 was performed. In total, 118 patients were included. For the classification of femur fractures in children, the AO Pediatric Comprehensive Classification of Long-Bone Fractures (AO-PCCF) was applied. Results: Simple oblique fractures (32-D/5.1) occurred in most of the patients. Patients with simple oblique fractures were significantly younger compared to patients with simple transverse (32-D/4.1) or multifragmentary (32-D/5.2) fracture type according to the AO Pediatric Comprehensive Classification of Long-Bone Fractures. Most patients were treated with elastic stable intramedullary nailing (68 patients, 58%). Although children treated with elastic stable intramedullary nailing were older than those treated conservatively (25%, *n* = 29, mean age 1.5, median age 1.0), the children in the elastic stable intramedullary nailing group were comparatively young (range 1–12 years, mean age 5.4, median age 5). A total of 32 children below the age of 6 years were treated with elastic stable intramedullary nailing. Complications were more frequent in patients with overhead extension (50%) compared to conservative treatment with a spica cast (17%) or elastic stable intramedullary nailing (15%). Conclusions: Elastic stable intramedullary nailing therapy was associated with a low complication rate and was, therefore, a safe and frequently used treatment strategy in diaphyseal femur fractures with satisfactory results, even though the age groups were expanded in favor of younger patients.

## 1. Introduction

Following the forearm and tibia, the femur is the most common fracture location in children, accounting for 2% of all pediatric fractures [1,2]. Nevertheless, femoral fractures are the most common injury in children requiring hospitalization [3]. In non-overweight children aged 6–12 years with diaphyseal femur fracture, elastic stable intramedullary nailing (ESIN) is the gold standard [4]. It has been demonstrated that the treatment of diaphyseal femur fractures in children by ESIN resulted in fewer economic and social side effects compared to alternative therapy strategies, including spica cast [5] and external fixation [6]. However, complication rates after ESIN have been reported in up to 80% of unstable and 50% of stable fractures [7,8,9]. Painful implants occurred in 20%, whereas prominent implants occurred in 12% of femoral fractures treated with ESIN [10]. Besides pain, protruding flexible intramedullary nails caused delayed knee mobilization [9]. Angulation and shortening were common complications that occurred with the ESIN treatment of unstable fractures [8]. Intraoperative complications such as the puncture of the opposite cortex during nail insertion occurred more frequently with stainless steel nails compared with titanium nails [11]. Risk factors associated with complications of ESIN therapy are inexperienced surgeons, greater patient age and weight and unstable fracture patterns [12,13,14]. Furthermore, biomechanical studies revealed that a ratio of the ESIN diameter and the canal diameter of 80% is optimal regarding the maintenance of reduction. Correspondingly, biomedical studies of the length of stable fractures showed that reduction stability is improved by a higher nail-to-canal ratio, whereas the restoration of the alignment was improved with a lower ratio [15]. In fractures that are unstable under axial loading as well as in complex fractures, a higher nail-to-canal ratio is associated with improved torsional and compression stability [15]. Additionally, stability is influenced by the insertion method, the fracture location, the three-nail versus two-nail model [16], antegrade versus retrograde nailing [17] and the all-lateral entry-technique versus the combined technique [18]. However, the treatment algorithm for diaphyseal femoral fractures in those of pediatric age is continually adjusted partly because of patient needs. Treating femur fractures in pediatric age by a locking plate seems to be a safe procedure with good functional outcome. Nevertheless, it is associated with a more invasive approach, and it is a more time-consuming procedure compared to ESIN [19]. Furthermore, conservative treatment needs to be clearly indicated, as shown by Maccagnano et al., showing that, when determined parameters such as the Cast Index are applied, future failure can be reduced when switching to operative treatment in time [20]. Overall, with regard to bone healing, one must always be aware that conservative treatment and therapy with ESIN induce indirect bone healing with clearly recognizable callus formation in the X-ray images, since only relative stability is induced by plaster or ESIN. In contrast, intramedullary nail osteosynthesis or plate osteosynthesis provide absolute stability, resulting in significantly less callus formation [21].

We hypothesize that ESIN is a feasible and safe procedure for femur fracture fixation, even in children below the age of 6 years, with respect to the duration of hospitalization, duration of callus formation and complications. It therefore represents an alternative to conservative therapy in younger children. Moreover, we hypothesize that, even in children younger than 3 years, ESIN can be a successful and safe method for the treatment of femoral shaft fractures. We further hypothesize that fracture type and treatment strategy are age-dependent and that the treatment strategy is dependent on the dislocation angle and angulation.

## 2. Materials and Methods

### 2.1. Design

Ethical approval was obtained from the local Ethics Board (No. 44/18). We performed a retrospective chart review of all patients <18 years with femur fracture treated at a Level I Trauma Center in Germany between 2005 and 2017. Patients were identified in the local picture archiving and communication system (PACS). Inclusion criteria were pediatric patients aged 0–17 years who were admitted to the hospital with traumatic femoral shaft fracture. Exclusion criteria were an age ≥18 years, a pathologic fracture and metabolic/genetic bone disease. In total, 118 patients were included in the retrospective analysis. Charts were reviewed to obtain demographic information, AO Pediatric Comprehensive Classification of Long-Bone Fractures (AO-PCCF) fracture type, treatment strategy and outcome with regard to callus formation and complications. Age groups were defined and adapted, according to Loder et al., as infant/toddler (<2 years), early childhood/preschool (age 2–5 years), middle childhood/elementary school age (age 6–12 years) and adolescents/middle and high school age (age 13–17 years) [3].

The classification of femur fractures in children was performed by applying the AO-PCCF of the AO Compendium of Fracture and Dislocations [22,23].

### 2.2. Treatment Strategies

The treatment strategies analyzed in the present study included conservative methods, invasive methods such as ESIN, intramedullary nail (IMN) and open reduction and internal fixation (ORIF) with plate osteosynthesis and an external fixator. The standard procedure of all treatment groups for the X-ray follow-up includes the initial X-ray and an X-ray after casting or osteosynthesis, followed by an X-ray after two weeks. The last X-ray was performed either after cast removal or after hardware removal. Since this was a retrospective data analysis, all p-values must not be interpreted in a confirmatory manner. Thus, no adjustment due to multiple hypothesis testing was required and performed, respectively. Decision making for operative treatment is either based on the stability of the fracture, the extent of displacement or the age.

### 2.3. Statistics

Data were analyzed using GraphPad Prism Version 7.0 (GraphPad Software Inc., San Diego, CA, USA). In the case of two groups, a non-paired student’s t-test was applied. In the case of three or more groups, a one-way Analysis of Variance (ANOVA) followed by Tukey’s multiple comparison test were used. *p* ≤ 0.05 was considered statistically significant.

## 3. Results

### 3.1. Demographics

**Figure 1:**
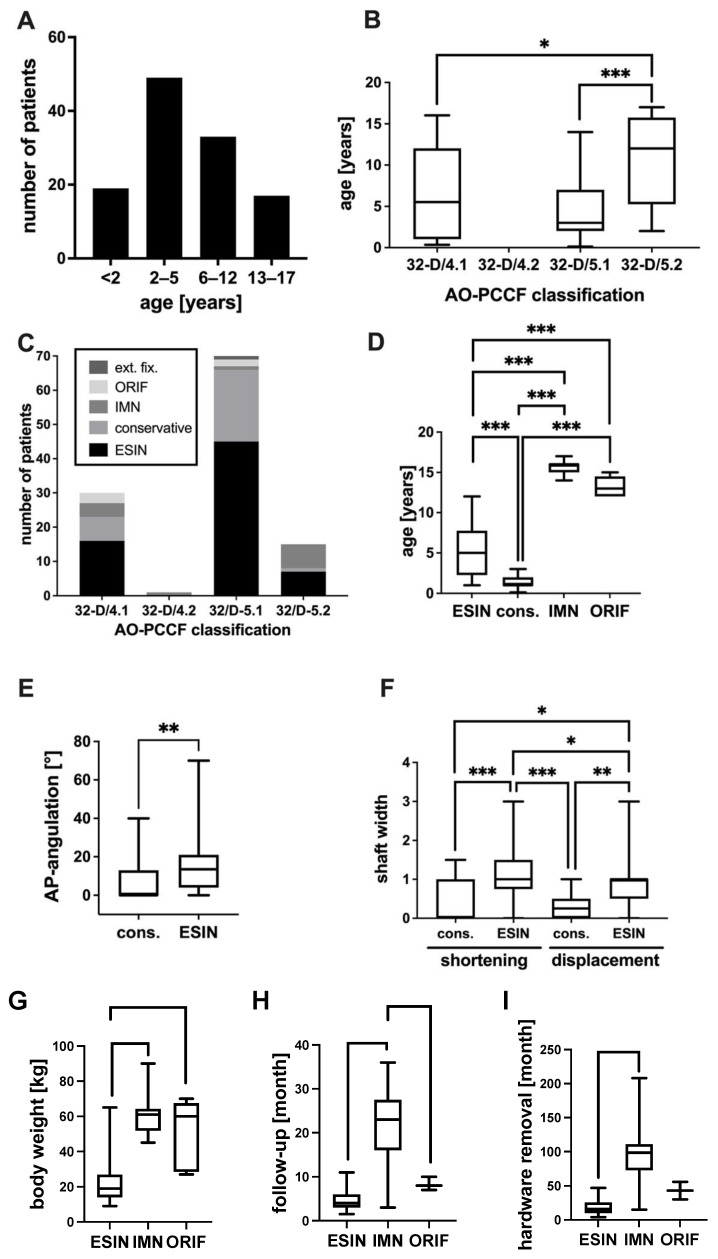
(**A**) Number of patients in the respective age groups of <2, 2–5, 6–12 and 13–17 years. (**B**) Patient age for the different fracture types: 32-D/4.1, 32-D/4.2, 32-D/5.1 and 32-D/5.2, according to the AO Pediatric Comprehensive Classification of Long Bone Fractures (AO-PCCF). (**C**) Numbers of patients and treatment strategies in the different AO-PCCF-fracture types: 32-D/4.1, 32-D/4.2, 32-D/5.1 and 32-D/5.2. (**D**) Angulation in the anterio-posterior X-ray projection in conservative-treated and ESIN-treated patients. (**D**) Patient age in the different treatment groups of flexible intramedullary nailing (ESIN), conservative (cons.), intramedullary nailing (IMN) and open reduction and internal fixation (ORIF) with plate osteosynthesis. (**E**) AP-Angulation in patients treated conservatively or with ESIN. (**F**) Shortening and fracture displacement in conservative-treated and ESIN-treated patients, depicted in the shaft width. (**G**) Weight of patients in respective treatment groups. (**H**) Duration of follow-up in months in the respective treatment groups. (**I**) Duration to hardware removal in months. Differences between groups were significant, as indicated: *: *p* < 0.05, **: *p* < 0.01, ***: *p* < 0.001.

In the present study, femoral shaft fractures occurred in 75 males (64%) and in 43 females (36%). The mean age of the children and adolescents was 6 years (median 4 years), ranging from 0 to 17 years. Femur fractures occurred most frequently in 2-year-old children (*n* = 23). The stratification to age groups adapted to Loder et al. revealed most children to be in the age group of early childhood/preschool age (2–5 years), followed by that of middle childhood/elementary school (6–12 years) (Figure 1A).

### 3.2. Diaphyseal Femur Fracture Type

The fracture type was classified according to the AO-PCCF in 32-D/4.1, 32-D/4.2, 32-D/5.1 or 32-D/5.2. A simple oblique fracture (32-D/5.1) occurred in most of the patients (*n* = 71, 60%), with the fracture line most frequently observed in the mid-shaft. A simple transverse fracture (32-D/4.1) was the second most common fracture type (25%), mainly located in the mid-shaft position. A multifragmentary oblique fracture (32-D/5.2) occurred in 14% of patients; here, again, the mid-shaft position was the dominant fracture localization. Multifragmentary transverse fractures (32/D-4.2) were rare and were found in the present study in only one patient. The fracture type correlated with the patient age. Patients with simple oblique fractures (32-D/5.1) and simple transverse (32-D/4.1) were significantly younger than those with multifragmentary oblique fractures (32-D/5.2) (Figure 1B).

### 3.3. Therapy

**Figure 2:**
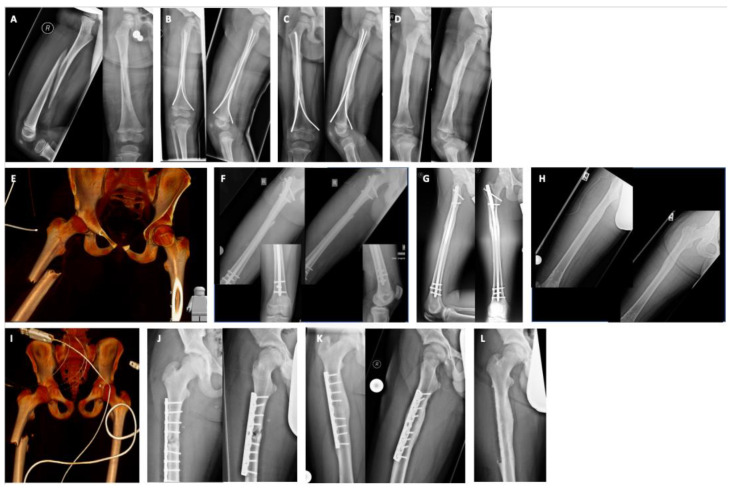
Different treatment strategies in children with femur fracturea. X-ray series demonstrating fracture type, treatment and bone healing process. (**A**–**D**): One-year-old boy twisted the leg while playing: 32-D/5.1 (**A**), ESIN (**B**) with secondary bone healing (**C**) and after hardware removal (**D**). (**E**–**H**): Sixteen-year-old boy after traffic accident with collision (moped against car): 32-d/4.1 (**E**), IMN (**F**) with mainly primary bone healing (**G**) and after hardware removal (**H**). (**I**–**L**): Twelve-year-old boy after traffic accident with collision (bicycle against car): 32-D/4.1 (**I**), ORIF (**J**) with mainly primary bone healing (**K**) and after hardware removal (**L**).

Treatment strategies included conservative methods, such as spica cast and overhead extension, as well as operative methods such as ESIN, IMN, ORIF or an external fixator. Most of the patients were treated by ESIN (68 patients, 58%), which was followed by conservative treatment (29 patients, 25%) by spica cast alone (19 patients) or overhead extension, which was followed by spica cast (10 patients). IMN was performed in 13 patients (11%), ORIF was performed in 5 patients (4%) and an external fixator was performed in 3 patients (3%). One patient treated by ORIF, six patients treated by IMN and two patients treated by ESIN were initially stabilized with an external fixator. Only three patients were treated with an external fixator alone. The therapy strategy correlated with the age (Figure 1D). Conservative therapy was performed in younger children compared to ESIN, IMN and ORIF. Furthermore, ESIN was performed in younger children compared to IMN and ORIF (Figure 1D). Even 16 patients younger than 3 years were treated with ESIN successfully without any complications (Figure 2A–D).

Moreover, treatment strategies were correlated with the fracture type. ESIN was the preferred therapy in simple fractures (32-D/4.1, 32-D/5.1), while the second most common therapy in such fractures was conservative treatment. In contrast, in multifragmentary oblique fractures (32-D/5.2), ESIN and IMN were used equally often (Figure 1C). Furthermore, we analyzed the fractures, regarding length-stability and length-instability, treated conservatively with ESIN, IMN and ORIF (Table 1).

Additionally, as expected, preoperative fracture dislocation and angulation were correlated with treatment strategy (Figure 1E,F). Axis deviation (Figure 1E) as well as malalignment and shortening (Figure 1F) were more frequent in patients treated by ESIN compared to those treated with conservative treatment strategies.

In order to make the investigated groups comparable, the body weight was matched according to the applied therapy (Figure 1G). Children treated with ESIN showed a significantly lower body weight compared to children with IMN or ORIF. Moreover, we analyzed the follow-up period in the different treatment groups (Figure 1H). The mean follow-up in the ESIN-group was 4.4 months compared to 21.2 months in the IMN-group and 8.3 months in the ORIF group. The duration to hardware removal was significantly longer in patients treated with IMN compared to patients treated with ESIN (Figure 1I).

With the aim of presenting treatment groups in terms of the fracture type, treatment and bone healing process, we present the corresponding case series of three treatment options (ESIN, IMN, ORIF). As an example of secondary bone healing, we demonstrate the relative stability of osteosynthesis by ESIN (Figure 2A–D) and the predominant primary bone healing with absolute stability by IMN (Figure 2E–H) and ORIF (Figure 2I–L).

### 3.4. Hospitalization and Outpatient Visits

**Figure 3:**
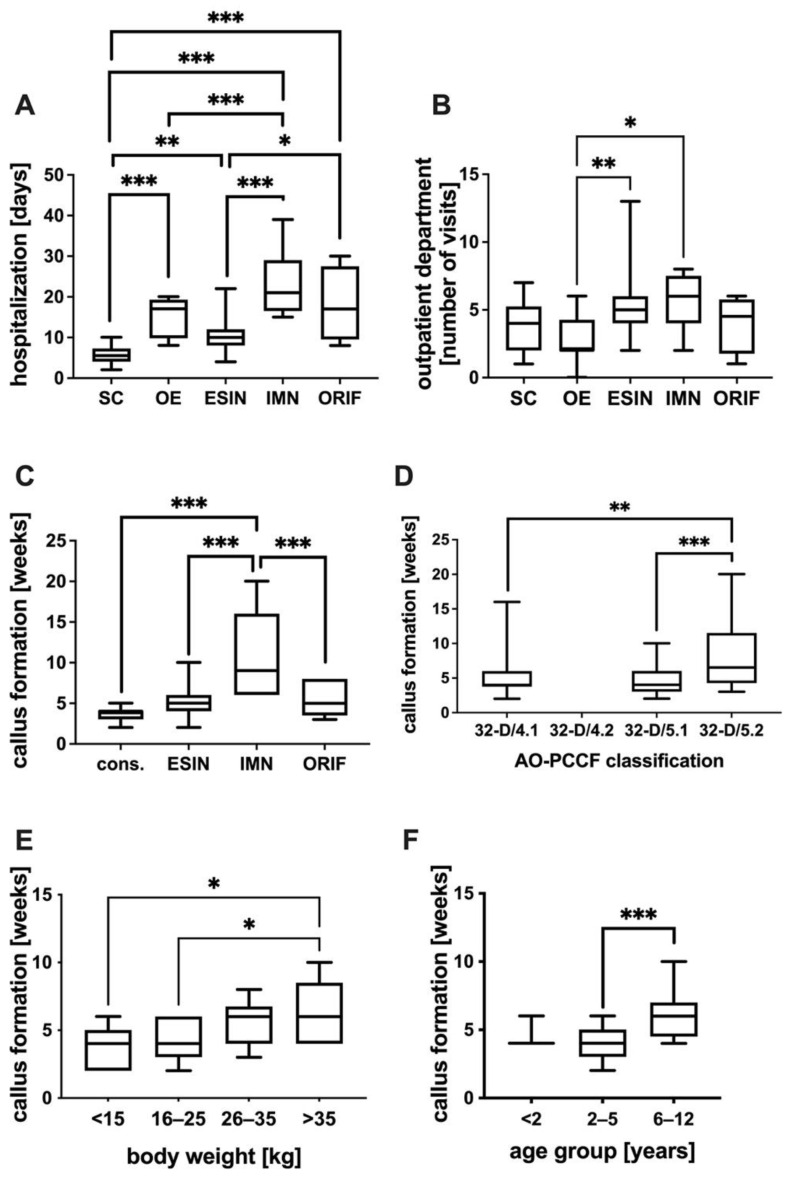
(**A**) Duration of hospital stay in days for the different treatment strategies of spica cast alone (SC), overhead extension followed by spica cast (OE), flexible intramedullary nailing (ESIN), intramedullary nailing (IMN) and open reduction and fixation (ORIF) with plate osteosynthesis. (**B**) Number of visits to the outpatient department among patients with the different treatment strategies of SC, OE, ESIN, IMN and ORIF. (**C**) Duration to callus formation in weeks under different treatment regimens: conservative treatment (cons.), flexible intramedullary nailing (ESIN), intramedullary nailing (IMN) and open reduction and internal fixation (ORIF) with plate osteosynthesis. (**D**) Duration to callus formation in weeks in the different fracture types: 32-D/4.1, 32-D/4.2, 32-D/5.1 and 32-D/5.2, according to the AO Pediatric Comprehensive Classification of Long Bone Fractures (AO-PCCF). (**E**) Duration of callus formation in weeks in the ESIN group in different body weight groups in kg. (**F**) Duration of callus formation in the ESIN group in weeks in different age groups. Differences between groups were significant, as indicated: * *p* < 0.05, ** *p* < 0.01, *** *p* < 0.001.

The duration of the hospital stay was significantly shorter in spica cast (SC) alone-treated patients compared to ESIN-trated ptients, followed by ORIF-, overhead extension (OE)- and IMN-treated patients. Furthermore, patients with ESIN had a shorter hospital stay compared to those with IMN or ORIF (Figure 3A). Besides OE resulting in fewer outpatient visits compared to ESIN and IMN, no difference in the number of outpatient department visits concerning the treatment groups can be found (Figure 3B).

### 3.5. Callus Formation

Callus formation was detected by X-ray. The duration of callus formation was significantly increased in IMN patients compared with ORIF, ESIN or conservative therapy patients (Figure 3C). The duration of callus formation was significantly shorter in patients with simple oblique or transverse fractures (32-D/5.1, 32-D/4.1) compared to those with multifragmentary oblique fractures (32-D/5.2) (Figure 3D). Subgroup analysis of ESIN-treated patients revealed a shorter time period to callus formation in lower-body-weight groups (Figure 3E) and in lower-age groups (Figure 3F).

### 3.6. Complications

The analysis of adverse events in patients with diaphyseal femur fracture identified dislocation and the loss of reduction as the most frequently occurring complications, followed by skin irritation. Rare complications were infection and ESIN overlap or malposition. During overhead extension, complications were most frequently observed (50%) in the present study (Table 2).

### 3.7. Concomitant Injuries

Concomitant injuries were observed in 29 patients. This included 11 IMN-treated patients, 12 ESIN-treated patients, 3 ORIF-treated patients and all patients treated with an external fixator alone. The most frequently observed injuries were traumatic brain injury, additional fractures and soft tissue injury followed by lung contusion and abdominal trauma. In patients treated conservatively, no additional injuries were recorded. The mean age of patients with concomitant injuries was 11 years (median 12.5, 2–17 years).

Subgroup analysis of patients below three years and between three and six years.

The subgroup analysis of patients under three years of age and between three and six years of age who were treated either conservatively or with ESIN showed the following: 29 patients were treated conservatively, of which 25 patients were younger than three years. In total, 45 patients treated with ESIN were six years of age and younger, of which 16 patients were younger than three years of age. The predominant AO-PCCF fracture type in these patients was 32-D/5.1. The time to callus formation was significantly shorter in children younger than three years of age when treated conservatively in comparison to children treated with ESIN. As expected, the follow-up duration was longer in patients treated with ESIN. All patients younger than 3 years who obtained ESIN showed no complications (Table 3).

## 4. Discussion

The purpose of this study was to elucidate diaphyseal femur fractures in children in order to evaluate ESIN treatment with regard to the duration of hospitalization, the duration of callus formation and the frequency of complications.

First, we analyzed the demographics of our patient cohort. In the present study, 64% of patients were male and 36% were female. This distribution confirms those of earlier studies, which recorded that male children are more frequently affected by femur fractures [3,24]. Overall, children below the age of six years (*n* = 48, 40%) were recorded most frequently with diaphyseal femur fractures; in our study, 23 children were two years old. These findings are in accordance with the literature. In a recently published epidemiologic study, 2-year-old children displayed the highest proportion of femur fractures [25]. Beyond the previous findings, we characterized the diaphyseal femur fractures in more detail, applying AO-PCCF classification. Patients with AO-PCCF fracture type 32-D/5.1 (simple oblique fractures) and 32-D/4.1 (simple transverse) were significantly younger compared to patients with 32-D/5.2 (multifragmentary oblique fractures). Simple oblique and simple transverse fractures were most frequently treated with ESIN, followed by conservative treatment. Patients with ESIN were significantly older compared to those with conservative treatment.

In order to answer the question of whether ESIN treatment is a safe and feasible procedure, even in children aged below 6 years, we analyzed the duration of hospitalization, the duration of callus formation and the frequency of complications in the respective treatment strategies. The duration of hospitalization was significantly longer in patients treated with ESIN or OE compared to patients treated with spica cast. A recently published financial analysis compared spica casting to flexible intramedullary fixation in children with closed femur fractures [26]. Here, ESIN was likewise associated with a longer in-hospital stay, higher hospital charges, a longer follow-up and more out-patient visits compared to spica casting [26]. In contrast, in the present study, there was no difference in the number of visits in the outpatient department between ESIN and SC, IMN or ORIF, it only showing fewer outpatient visits in OE compared to ESIN and IMN.

Interestingly, with regard to callus formation, there was no significant difference between conservative and ESIN treatment. An analysis of ESIN-treated patients displayed shorter time periods to callus formation in the age group of 2–5-year-old children compared to the 6–12-year-old patients. However, the duration of callus formation in the 2–5-year-old children was not significantly shorter than that in children aged <2 years. This might be due to variable X-ray intervals and a low number of cases. However, the bone consolidation process was prolonged in multifragmentary oblique fractures (32-D5.2) compared to both simple oblique fractures (32-D/5.1) and simple transverse (32-D/4.1) fractures. Moreover, as expected, the duration of callus formation was longer in patients with a higher body weight and in older children. In the literature, no differences in the clinical or radiographic outcomes in school-aged children or in 4–5-year-old children have been reported between femoral fractures treated with ESIN and spica casting [4,26].

The limitation of the present observation is that, due to the retrospective character of the study, we do not have sufficient complete information on the clinical outcome, such as full-weight bearing or functional parameters. Moreover, all p-values presented need to be interpreted carefully in a hypothesis-generating manner.

Complications of the flexible intramedullary nail fixation of pediatric femoral fractures have been shown to correlate with the fracture type [8,12,27]. In the present study, the fracture type and treatment strategy were age-dependent. However, ESIN treatment was applied in 38 patients below the age of 6 years, whereas children with conservative treatment were 3 years of age or younger. In the present study, complications such as skin irritation, infection, dislocation and loss of reduction were very rare and occurred in 15% of patients treated with ESIN and in 17% of patients treated conservatively, and it occurred most frequently in children with overhead extension (50%). This is in contrast to published complication rates after ESIN of up to 80% in unstable fractures and 50% in stable fractures [7,9]. Furthermore, in a subgroup analysis of children aged below 3 years with ESIN treatment, no complications occurred in all 16 patients, whereas six complications (24%) occured in the corresponding ESIN group (patients aged 3 years).

In our study, the complication rates of ESIN and conservative therapy were approximately the same, although children with conservative treatment were significantly younger compared to patients treated with ESIN. In the literature, children older than 11 years had a worse outcome compared to younger children [12]. Furthermore, the outcome of femur fractures in children was weight-dependent. A poor outcome was significantly increased in children who weighted >49 kg [12]. Moreover, in overweight children, the incidence of femur fractures was increased [28,29,30].

## 5. Conclusions

This study addressed the demographic and etiologic characteristics of diaphyseal femur fractures in children, as well as the AO-PCCF classification. Our results confirmed the findings from the literature regarding demographics and hospitalization. Callus formation was dependent on the fracture type, treatment strategy, age and body weight. Moreover, the present evaluation showed that the fracture type and treatment strategy were age-dependent. The treatment strategy was also dependent on the dislocation. In contrast to the literature, our results highlighted low rates of adverse events in ESIN-treated patients, even in younger children. Furthermore, we showed that ESIN can be used successfully and safely in patients under 3 years of age. Thus, it is conceivable to expand the indication and partially replace more complication-prone procedures such as overhead extension and spica.

## Figures and Tables

**Table 1:** Portion of length-stable and length-instable fractures of the femur shaft in the different treatment groups: conservative, flexible intramedullary nailing (ESIN), intramedular nail (IMN) and ORIF, external fixator (EF).

Stability	Conservative	ESIN	IMN	ORIF	EF
Length-stable	4 (14%)	5 (16%)	1 (8%)	0 (0%)	0 (0%)
Length-instable	25 (86%)	26 (84%)	12 (92%)	5 (100%)	3 (100%)

**Table 2:** Summary of adverse events in the different treatment groups: flexible intramedullary nailing (ESIN), spica cast (SC) alone and after overhead extension (OE) and during overhead extension.

Adverse Events	ESIN *n* = 66	SC *n* = 29 (After OE *n* = 10)	OE *n* = 10	IMN *n* = 13	ORIF *n* = 5
skin irritation	1	2 (2)	2	-	-
infection	2	2	0	-	-
dislocation/loss of reduction	4	-	3	2	1
ESIN overlap	2	-	-	-	-
ESIN malposition	1	-	-	-	-
total	10 (15%)	4 (14%)	5 (50%)	2 (15%)	1 (20%)

**Table 3:** Subgroup analysis of patients 6 years and younger. Anterior-posterior (AP), AO Pediatric Comprehensive Classification of Long-Bone Fractures (AO-PCCF), standard deviation (SD), male (m) and female (f), number of patients (*n*).

	Conservative		ESIN	
	0–<3	3–6	0–<3	3–6
age [years]				
*n*	25	4	16	29
mean [SD]	1.16 [0.75]	3.0 [0]	1.813 [0.40]	4.414 [1.15]
median [min/max]	1 [0/2]	3 [3/3]	2 [1/2]	4 [3/6]
gender [m/f]	14/11	4/0	7/9	20/9
AO-PCCF fracture type [n]				
32-D/4.1	7	0	1	4
32-D/5.1	18	3	14	22
32-D/5.2	0	1	1	3
AP-angulation [°]				
*n*	21	4	14	22
mean [SD]	6.0 [10.13]	7.0 [8.25]	17.5 [18.11]	13.4 [11.83]
median [min/max]	0.0 [0/40]	6.0 [0/16]	16.0 [0/70]	12.0 [0/45]
displacement [shaft width]				
*n*	25	4	16	26
mean	0.36 [0.36]	0.50 [0.58]	0.66 [0.37]	0.78 [0.30]
median [min/max]	0.5 [0/1]	0.5 [0/1]	0.8 [0/1]	1.0 [0/1]
shortening [shaft width]				
*n*	25	4	16	26
mean [SD]	0.44 [0.56]	0.50 [0.58]	0.78 [0.45]	1.04 [0.71]
median [min/max]	0 [0/1.5]	0.5 [0/1]	1 [0/1.5]	1 [0/3]
callus formation [weeks]				
*n*	20	4	13	20
mean [SD]	3.45 [0.69]	4.25 [0.50]	4.62 [1.39]	4.35 [1.69]
median [min/max]	4 [2/4]	4 [4/5]	5 [2/6]	4 [2/8]
follow-up [weeks]				
*n*	26	4	16	27
mean [SD]	3.79 [2.42]	4.0 [0.82]	12.38 [9.70]	13.05 [6.49]
median [min/max]	4 [0/12]	4 [3/5]	10 [4/45]	12 [2/34]
complications n/%	6/24%	1/25%	0/0%	4/14%

## Data Availability

The data presented in this study are available on request from the corresponding author.
